# Dynamic and Ballistic Performance of Uni- and Bidirectional Pineapple Leaf Fibers (PALF)-Reinforced Epoxy Composites Functionalized with Graphene Oxide

**DOI:** 10.3390/polym14163249

**Published:** 2022-08-10

**Authors:** Pamela Pinto Neves, Ulisses Oliveira Costa, Wendell Bruno Almeida Bezerra, André Ben-Hur da Silva Figueiredo, Sergio Neves Monteiro, Lucio Fabio Cassiano Nascimento

**Affiliations:** Department of Materials Science, Military Institute of Engineering, IME, Rio de Janeiro 22290-270, Brazil

**Keywords:** pineapple fiber, ballistic performance, graphene oxide, bidirectional composites

## Abstract

Replacing synthetic fibers with natural ones as reinforcement in polymeric composites is an alternative to contribute to sustainability. Pineapple leaf fibers (PALF) have specific mechanical properties that allow their use as reinforcement. Further, graphene oxide (GO) has aroused interest due to its distinctive properties that allow the improvement of fiber/matrix interfacial adhesion. Thus, this work aimed to evaluate the ballistic performance and energy absorption properties of PALF-reinforced composites, presenting different conditions (i.e., GO-functionalization, and variation of fibers volume fraction and arrangement) through residual velocity and Izod impact tests. ANOVA was used to verify the variability and reliability of the results. SEM was employed to visualize the failure mechanisms. The Izod impact results revealed a significant increase in the absorbed energy with the increment of fiber volume fraction for the unidirectional configuration. The ballistic results indicated that the bidirectional arrangement was responsible for better physical integrity after the projectile impact. Furthermore, bidirectional samples containing 30 vol.% of GO non-functionalized fibers in a GO-reinforced matrix showed the best results, indicating its possible application as a second layer in multilayered armor systems.

## 1. Introduction

Protection against firearm projectiles, as well as fragments, and shrapnel is a matter of personal defense and safety, especially for police, military, and related services [1,2]. In this sense, bulletproof vests must have the ability to reduce fatalities. These vests are considered efficient in lessening body harm [2,3]. Among the main parameters for the survival of a combat system are mobility, protection, and firepower. Other factors, such as the design and materials selection, influence the ballistic performance of the vests. They aim to decrease trauma caused by projectile impact and enhance energy absorption characteristics while reducing total weight [3,4]. Over the past decades, different body armor systems have been developed to provide ballistic resistance against high-velocity impacts. Among those, the multilayered armor system (MAS) offers markedly adequate protection against high-velocity projectiles, such as 7.62 mm caliber [1,3].

In general, multilayered armor systems (MASs) are composed of at least two distinct layers. First, a hard ceramic front layer that deforms and shatters the projectile, mitigating the localized pressure imposed on the back plate. Then a second layer of a fiber-reinforced composite plate hinders the projectile movement after the ballistic impact, absorbing part of its kinetic energy. This composite structure can transfer the impact stress effectively in the transverse and longitudinal directions due to its long fiber reinforcements [4,5,6,7,8].

In terms of the MASs second layer, composites or laminates of high strength, low-weight synthetic fibers can be used, such as aramid (Twaron™ and Kevlar™) and ultra-high molecular weight polyethylene (UHMWPE) (Spectra™ and Dyneema™), which stand out as reinforcement in polymeric composites [1,9]. However, as a sustainable alternative, several natural lignocellulosic fibers (NLFs) have been investigated to replace synthetic fibers. Indeed, desirable characteristics, such as recyclability, biodegradability, and renewability, combined with lower cost and density to justify the choice of NLFs [10]. However, their properties are associated with natural variations in age, source of fiber in the plant, fiber size, cellulose content, and fiber strength [11], all of which depend on plant growth and cultivation conditions [12]. Thus, for their use in composites applied as MAS second layers, NLFs-specific supplied lot must always be previously characterized. Among these NLFs, the fique [9,13], ramie [14,15], guaruman [16], hemp [17], piassava [5], jute [18,19], mallow [18,19,20], sisal [21,22], kenaf [23,24], curaua [25,26], coconut [27,28], bamboo [29], sugar cane [30], and PALF [27,31] are worth highlighting.

Pineapple leaf fibers from the plant *Ananas comosus*, also known as PALF, were selected for this research. They contain about 70–82% cellulose, 6–12% hemicellulose, 5–12% lignin, and 1.1% residue in their composition [1,32,33,34]. According to Reddy and Yang [35], this higher cellulose content in the PALF might be related to the heavier fruit they must bear and to their lesser perishability. Additionally, these natural fibers present a low microfibrillar angle (14°), which, combined with the cellulose content, yields them better mechanical strength when compared to other natural fibers [1,32,33,34]. These positive aspects of PALF make them an excellent choice for the manufacture of polymeric composites, in addition to the sustainability aspect, considering that such fibers are agricultural residues [32,33,34,35,36].

The mechanical and chemical characteristics of the fibers, matrices, and their interface determine how the composite deforms and fractures. Furthermore, parameters, such as component geometry, also affect the composite’s ballistic impact response. Therefore, the present research investigated the structural behavior by varying the fiber orientation angle [1,37]. The layered architecture of composites significantly influences the impact behavior of a composite, assisting to determine whether such composite is suitable for potential application in personal ballistic protective equipment [37,38]. The ballistic approach is presented in the works of Reis et al. [16], Ribeiro et al. [17], and Neuba et al. [39] on polymeric composites reinforced with natural fibers. In those works, standalone ballistic tests with 0.22 caliber ammunition (level I—according to the protection level specification by NIJ 0101.04 [40]) were conducted to evaluate the absorbed energy and the limit velocity of compression molding manufactured composites.

The present research also investigated the effect of functionalization with graphene oxide (GO) in composites to improve their ballistic protection capability. Some studies in the literature conducted GO treatment in NLFs reinforced polymeric composites, namely piassava [41], jute [42], and sisal [43] fibers. In terms of ballistic applications, curaua [25] and ramie [44] fibers stand out. The literature [25,45,46,47] also reports that the incorporation of GO imparts improvement to the interfacial adhesion, due to the interaction of GO’s functional groups with the hydroxyl groups in the NLFs’ cellulose chains. Significant enhancements in tensile and flexural strengths, as well as impact resistance, ballistic penetration, physical integrity of composites, water resistance, and thermal stability optimized properties after functionalization, have been previously reported [10,48]. Thus, such treatment with GO demonstrates itself to be an effective method in improving the properties of the fiber/matrix interface, making it more efficient for impact energy dissipation [25].

Therefore, this work aims to evaluate for the first time the ballistic behavior of PALF-reinforced epoxy composites, with and without GO functionalization, in unidirectional and bidirectional fiber configurations through standalone tests using 45 caliber ammunition (level IIIA). It is also an objective to study the mechanisms of energy absorption through SEM analysis of samples after impact.

## 2. Materials and Methods

### 2.1. Materials

In the present study, three materials were used to manufacture the composite plates: PALF fibers, epoxy resin, and graphene oxide (GO). The PALF were donated by Embrapa, Cruz das Almas, Brazil. The resin was obtained from the company Epoxyfiber Ltd., Rio de Janeiro, Brazil. The GO was produced in collaboration with the Thin Films Laboratory at the Military Institute of Engineering—IME, Rio de Janeiro, Brazil).

The fiber preparation process involved brushing, cleaning, oven drying, and cutting into 12 and 15 cm in length, depending on fibers arrangement direction. The commercial resin, i.e., bisphenol A diglycidyl ether (DGEBA), was mixed with the hardener triethylenetetramine (TETA) in a stoichiometric ratio of 100 parts to 13. The GO was prepared using the Hummers and Offeman method modified by Rourke et al. [47], according to the procedure described in Lima et al. [48]. Since the concentration received was 7.3 mg/mL, a dilution was necessary to achieve the final GO concentration in the composites of 0.1 wt.%. Hence, GO was diluted to 0.2 mg/mL to proceed with incorporation into PALF fibers. Subsequently, the PALF fibers were immersed in the diluted GO solution under stirring and then conditioned in an oven at 60 °C for 24 h to evaporate the solvent [25]. The incorporation of GO into the epoxy matrix followed the method described by Costa et al. [46], in which the uncured resin and a GO/isopropyl alcohol suspension are mixed and dried in an oven at 60 °C to completely remove the alcohol. Sequentially, the composite was prepared with the addition of the hardener.

### 2.2. Composites Preparation

Plates of both PALF-reinforced epoxy composite and plain epoxy, as control, were fabricated in a 15 × 12 cm^2^ steel mold. Composite plates were manufactured by hand lay-up layers of aligned PALF, and then pouring still fluid DGEBA/TETA resin onto each layer. The layers’ assembly is explained in the upcoming Section 2.3. All plates were fabricated with 1 cm (tolerance of 1 mm) thickness.

The volume fractions of the PALF-reinforced composites followed the proportions of 10, 20, and 30 vol.%. For the manufacture of the composite, a hydraulic press SKAY, Rio de Janeiro, Brazil, was used, applying a load of five (5) tons for 8 h in the metallic mold. The curing time used was based on [49], in which it was concluded that the average time to achieve a transformed fraction of 95% of 20 vol.%-mallow fiber-reinforced epoxy was 240 min (4 h). Therefore, an 8 h curing time was selected to allow enough time to maximize the cured epoxy fraction.

### 2.3. PALF Assembly in Composite Plates

**Figure 1:**
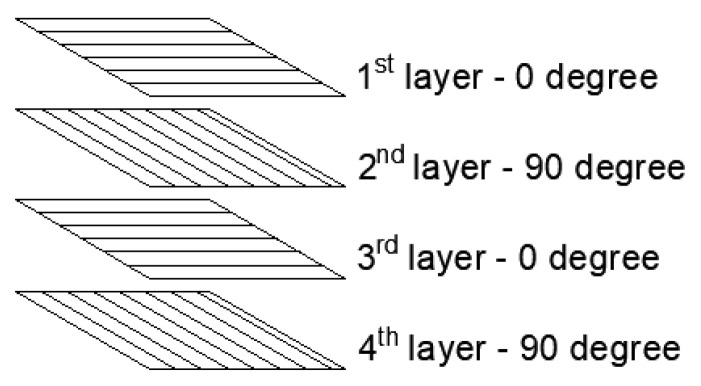
Schematic representation of sequential PALF layers.

Regarding the assembly of unidirectional and bidirectional plates, single-length 15 cm long fibers were arranged in the longitudinal direction in the unidirectional plates. As for the bidirectional plates, fibers with two distinct lengths were distributed in 4 layers, two 12 cm long fiber layers, and two with fibers of 15 cm in length. The layered distribution of bidirectional plates follows a similar pattern to the ones described by Chiu and Young [50] and Dimeski and Bogoeva-Gaceva [51], as shown in Figure 1.

Cross-orienting PALF layers, as shown in Figure 1, have the benefit of providing homogeneity in strength for both directions, similar to plywood structures used in the wood products industry for many decades. As indicated by Naghizadeh et al. [52], for high-velocity impact loading, such as that caused by fast-moving ballistic projectiles, plywood composites showed higher absorbed energy than solid wood with mostly unidirectionally aligned cellulose fibers.

### 2.4. Ballistic Tests

**Figure 2:**
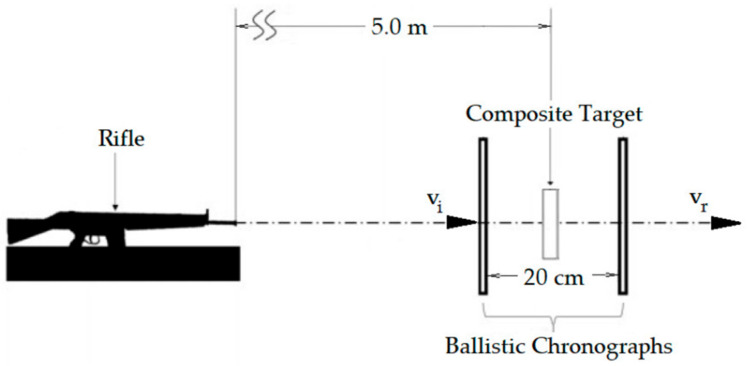
Detection system in the ballistic energy absorption test [16].

The ballistic test was carried out based on the NIJ 0101.04 [40] standard at the IME. The residual velocity test was selected to determine the energy absorbed by the different composites. This test was conducted using 45 caliber lead ammunition, with an estimated projectile mass of 14.4 g. The test was performed using an Airforce Texan pressure rifle 5 m away from the target and two ProChrono model Pal ballistic chronographs positioned 10 cm before and after the target (see Figure 2). This arrangement allows one to determine the variation in the projectile’s velocity, to quantify the absorbed energy through Equation (1) and the limit velocity through Equation (2), based on measured velocity values at impact and after projectile perforation:

(1) $$E_{abs}=\frac{1}{2}m\left(V_{0}^{2}-V_{R}^{2}\right)-E_{abs}^{*}\;\left({\;}^{*}from\;calibration\;shot\;with\;no\;sample\right),$$

(2) $$V_{L}=\sqrt{\frac{2}{m}E_{abs}}\;,$$

where, *E_abs_* is the energy absorbed, *V_L_* is the limit velocity, m is the projectile mass, *V*_0_ is the impact velocity (initial), and *V_R_* is the residual velocity (after perforation).

Five shots were executed for each group, except for groups 1 and 2, for which there was only 1 shot. The groups differ by their plate’s composition, fiber arrangement, and GO functionalization. The studied groups are labeled in Table 1. Obtaining *V_R_* was possible due to the composites being fully perforated.

### 2.5. Izod Impact Tests

The Izod impact test was performed on untreated PALF composites (NT) to determine the energy absorbed after the pendulum impact, in Joules per meter (J/m), based on the ASTM D256 standard [53]. Ten specimens were produced with dimensions of (63.5 × 12.7 × 10) mm^3^, from the composite plates of each group. The tests were carried out in a Pantec equipment (Rio de Janeiro, Brazil), model XC-50, with a 22 J pendulum. The specimen notches had a V-shaped 45° angle, a vertex radius of curvature of 0.25 mm, and a depth of 2.54 mm.

For the scanning electron microscopy (SEM) fractographic analysis, a model Quanta FEG 250 (FEI, Hillsboro, OR, USA) was used. The samples observed by SEM were extracted from the region around the hole resulting from the ballistic test. All samples were sputter-coated with gold.

## 3. Results and Discussion

### 3.1. Izod Tests

**Figure 3:**
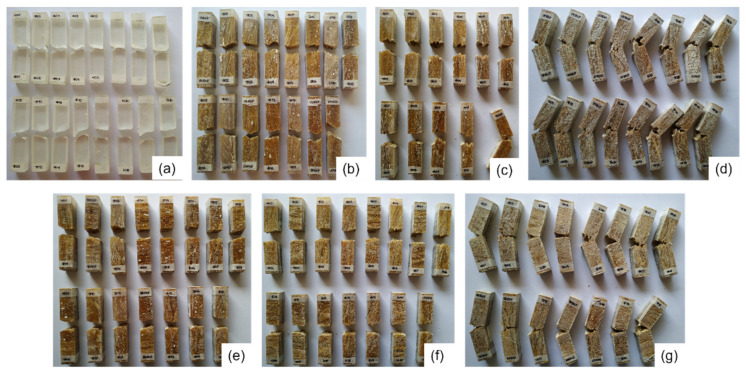
Groups of specimens containing 100% epoxy (**a**), unidirectional (UD) composites with 10% (**b**), 20% (**c**), and 30 vol.% of PALF (**d**); and bidirectional (BD) composites with 10% (**e**), 20% (**f**), and 30 vol.% PALF (**g**).

The samples after the Izod impact test are shown in Figure 3, in which all composites underwent complete rupture, except for the group containing 30 vol.% of fibers and for one sample of the group with 20 vol.% unidirectional PALF-epoxy.

**Figure 4:**
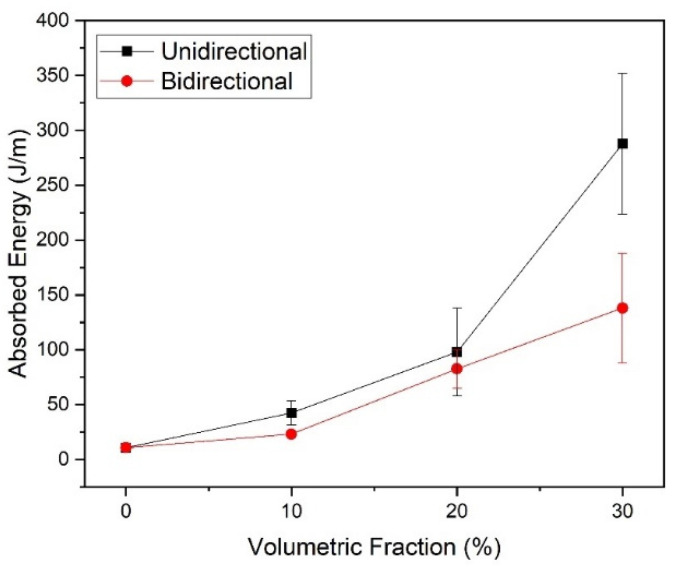
Izod impact absorbed energy vs. fiber reinforcement content of the unidirectional and bidirectional specimens.

Figure 4 shows the graph with the averages and standard deviations of these values related to the variation of the Izod impact resistance.

According to the results presented, it is possible to verify a relationship between the increase in the PALF volume fraction and the Izod impact energy. With the change from 10% to 30 vol.% of fiber, the energy absorbed after impact increases significantly, around 579% for unidirectional composites and 497% for bidirectional composites. Rahman et al. [54] also reported an increase in the impact strength with the addition of fiber content, however a drop of 19.30% was observed at further volume fractions, i.e., 60 vol.%.

This observed increase was expected due to the epoxy’s fragile behavior, in which the impact generated crack on unreinforced specimens propagates without restrictions until a complete fracture occurs. However, in the case of fiber-reinforced epoxy, the initial crack has its propagation blocked, leading to the migration of cracks to the fiber/matrix interface. Consequently, reinforced samples were not fully ruptured and bent after hammer impact due to the flexibility of the fibers [55]. This partial failure behavior indicates high tenacity imparted by the PALF reinforcement on the composites and implies a higher amount of energy absorbed if total failure was observed [27].

The PALF play a vital role in the impact resistance of composites since they interact with the formation and propagation of cracks in the matrix and function as a stress transfer medium [54]. It is worth noting that the standard deviation presented is due to the heterogeneous nature of any NLF, resulting in substantial dispersion of their reinforced composites’ properties [56]. Cellulose, hemicellulose, and lignin are the main constituents of NLFs, such as PALF, and their proportion in the fiber depends on the age, source of the fiber, and the extraction conditions used to obtain the fibers [35]. Hemicellulose usually acts as a filler between cellulose, contributing little to the stiffness and strength of fibers. In a different manner, lignin acts as a glue in the fiber structure, stiffening the cell wall and influencing the flexibility of the fibers. In high-cellulose content fibers, such as PALF (i.e., 80%), cracks propagate through the cell interface, causing intercellular fracture without the removal of microfibrils. In contrast, in fibers with lower cellulose content cracks propagate through the cells and result in intracellular fracture with microfibrillar pullout [35].

ANOVA analysis was performed to verify the reliability of the results. However, those referring to partially ruptured specimens should not be considered and consequently not used statistically, according to item 5.8 of ASTM D256 [53].

In fact, the statistical test indicated that the calculated F (31.13) > critical F (2.58), rejecting the hypothesis that the means are equal with a confidence level of 95%. Therefore, changing the volumetric fraction of PALF in epoxy matrix composites influences the Izod impact energy. In addition, the Tukey test was applied to compare means to verify which group presents the best results in terms of Izod impact energy. Table 2 shows the Tukey analysis results, in which the absolute mean difference (ADM) between the groups are shown, with the values above the honestly significant difference (HSD) highlighted in bold to indicate the difference.

The ADM values may be either positive or negative, based on the direction of which the significant change was achieved, i.e., increase or decrease in the mean values compared to the reference.

Based on these results, uni- and bidirectional composites reinforced with 20 vol.% PALF presented the best performances and the most significant difference in terms of the value of Izod impact energy.

In order to compare the results found with those found in the literature, Table 3 presents Izod impact energy values for polymeric composites with different NLFs. In this table, one can see that the values for the 30 vol.% PALF-reinforced epoxy composites have considerable variation between the various papers.

### 3.2. Ballistic Tests

**Figure 5:**
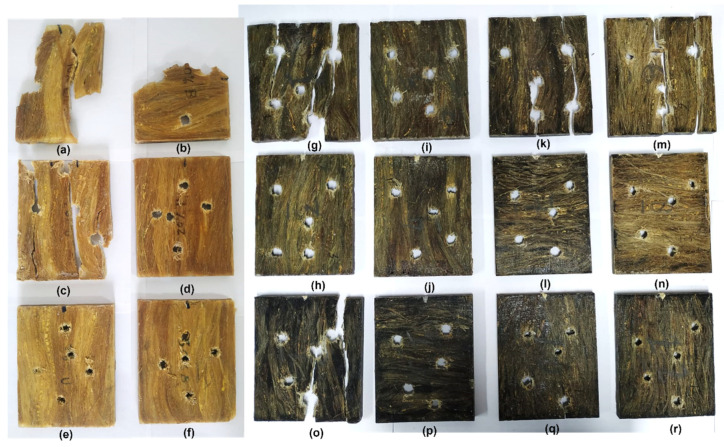
Aspect of the composite plates after ballistic test for each group: 10UD (**a**), 10BD (**b**), 20UD (**c**), 20BD (**d**), 30UD (**e**), 30BD (**f**) for PNT/ENT; 20UD (**g**), 20BD (**h**), 30UD (**i**), 30BD (**j**) for PGO/ENT; 20UD (**k**), 20BD (**l**), 30UD (**m**), 30BD (**n**) for PNT/EGO; and 20UD (**o**), 20BD (**p**), 30UD (**q**), 30BD (**r**) for PGO/EGO.

Regarding the ballistic test result, the samples perforated after the test are shown in Figure 5.

Table 4 presents the values referring to the averages of absorbed energy (Equation (1)) and limit velocity (Equation (2)).

It is important to emphasize that the residual velocity test was performed in the subsonic region, with velocities below 300 m/s, using a pressure rifle and a 45 caliber projectile [60]. The shock wave appears in the supersonic region and is not perceived in the subsonic regime [61]. Considering that, the classical field theory can be applied in order to convert the absorbed energies, based on the projectile’s mass. This physical theory portrays the interaction of systems made up of particles and fields, having as basic entities a set of space and time functions taken by the field equations [62,63]. In practical terms, the field is the velocity difference in magnitude while the particle is the projectile. Furthermore, this requires invariance in actions and equations of motion for a general solution containing an arbitrary scalar function of space–time coordinates [63,64,65].

In this context, the energy absorbed by a body follows a quadratic regime with a small variation in the parabolic representation of velocity (x-axis) versus energy (y-axis), with good tolerance. By considering the same projectile, it is possible to obtain the same energy value between two velocities in any region of the parabola. Thus, to compare the absorbed energy data reported in the literature regarding different projectiles, it becomes possible to convert the energies absorbed by the respective masses by dividing them. From that assumption, the absorbed energy of the present work was estimated considering the data presented in the NIJ 01.01.04 standard [40] on the calibers and their respective masses, as shown in Table 5. Table 6 illustrates the data found in the literature.

Comparatively, considering the values of absorbed energy achieved for the different groups of composites in the present study, the 30BD-PNT/EGO group, which reached the highest *E_abs_* value, was superior to the epoxy—PALF (30 vol.%) and polyester—curaua fiber (20 vol.%) composites corresponding to level III, and to aramid fabric, corresponding to level II. Considering the percentage of absorbed energy, the results found are adequate, since higher percentages mean higher kinetic energy dissipation through mechanisms, such as the delamination between layers, elastic deformation of the composite, shear of the layers, tension at rupture of the fibers, and brittle fracture of the epoxy matrix [19,20].

Thus, regarding the NIJ [40] classification of protection systems as levels I, II, and III, it is unfeasible to compare the present results with the literature data since none of the studies assessed level IIIA ammunition, which provides significant protection against firearms. In addition, it is worth mentioning that, in terms of the MAS performance, the energy absorption capacity of an individual component does not display the combined performance as a function of the synergistic effect. It indicates, however, the suitability for ballistic use [27].

For the ballistic tests, ANOVA was performed on the data to verify the reliability of the results. Tukey’s test was necessary given that the critical F (1.72) is smaller than the calculated F (2.22), thus rejecting the null hypothesis, for which the mean values are equal. Of the 190 combinations, 7 were significantly different. These are presented in Table 7, which refers to the ADM as higher than the reference value found by the test.

Thus, the treatment that most influenced the energy absorption of the composites was the one related to the addition of GO in the epoxy matrix (PNT/EGO). It is important to note that the composites with higher reinforcement percentages retain their physical integrity, unlike the 10UD and 10HD PNT/ENT groups and the 20UD composite plates, which fragmented after the shots. This fragmentation may be associated with the relative fragility of the epoxy matrix [5]. The 20UD plates of the PGO/ENT, PNT/EGO, and PGO/EGO groups were also fragmented, corroborating the influence of the bidirectional layers on the physical integrity of the composites, followed by the fiber volume. The fragmentation of the 30UD-PNT/EGO plate was an unexpected behavior, given the higher volumetric fraction and the coating with GO. Other studies report that an increase in fiber-epoxy interfacial adhesion is associated with GO functionalization, which in turn influences the physical integrity of the plates, a condition necessary for personal protective vests [10,46].

One of the analyses carried out in the study by Sundaram et al. [68] was the influence of fiber orientation on composite performance. They indicated that the bidirectional configuration provided better results due to the bending flexibility along the direction of bullet penetration, which was the reason for the higher absorption of the projectile kinetic energy compared to other fiber configurations. A similar observation was made in the works by Kumrungsie et al. [69]. The study described a computer simulation of the ballistic impact in polymeric composites with a bidirectional fiber arrangement and showed that the bidirectional plate absorbs more energy, resulting in a lower residual velocity of the penetrated projectile. However, despite the energy absorbed per area being relatively large, Dimeski et al. [51] drew attention to the disadvantage of crossing points in this arrangement. In the unidirectional orientation of the fibers, due to the lack of crossing points, that is, due to the inferior extension of the reflective impact wave. In fact, the ballistic impact wave is transmitted to a larger composite area, thus being able to present a superior performance.

In the present study, the functionalization of GO in the resin resulted in a 19.54% increase in absorbed energy. Comparing the untreated group (30BD-PNT/ENT) with the groups of the same fiber configuration and volume, varying only the treatment with GO, i.e., 30BD-PGO/ENT and 30BD-PGO/EGO, there was a decrease of 11%, 39% and 1.11%, respectively. In terms of the 30BD-PNT/EGO group, there was an increase of 36.19%.

Pereira et al. [44] studied the ballistic performance of composites, adding 0.5 wt.% of GO to the epoxy matrix reinforced with 30 vol.% of ramie fabric, reporting that the ballistic tests showed an increase in absorption energy, which was related to the presence of GO in the epoxy matrix. In the study by Silva et al. [65], the ballistic test was performed on the aramid fabric with and without GO treatment. The coating with GO showed a significant improvement in the absorbed energy of up to 50% when compared to the uncoated aramid fabric, due to the higher friction between the fibers. Therefore, GO functionalization in composites promotes an enhancement in energy absorption as a result of the interfacial interactions.

Despite the variability of the composite’s properties with the introduction of an NLF, literature suggests that when PALF and other natural fiber-reinforced composite plates are applied in an MAS they effectively stop bullet penetration and meet the NIJ back-face signature (BFS) depth criterion [16,31], offering protection in personal bullet proof vests.

### 3.3. SEM Analysis

**Figure 6:**
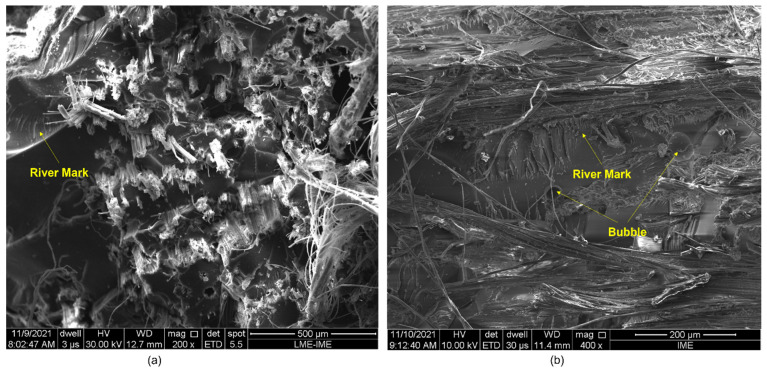
Fracture surface of a composite with 10 vol.% of fibers (PNT/ENT) arranged: unidirectionally, at 200× magnification (**a**) and bidirectionally, at 400× magnification (**b**).

**Figure 7:**
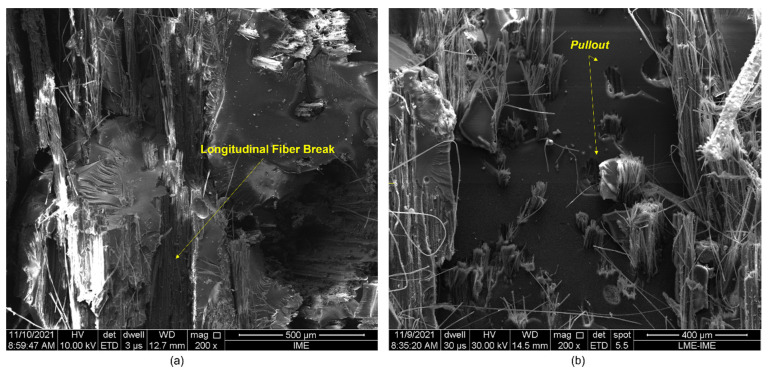
Fracture surface of a composite with 20 vol.% of fibers (PNT/ENT) arranged: unidirectionally (**a**) and bidirectionally (**b**), at 200× magnification.

SEM analysis was performed on the samples after ballistic impact to verify the active failure mechanisms and better understand the energy dissipation. Figure 6 and Figure 7 show the untreated composites’ fracture surfaces, depicting the fibers and the matrix. The samples containing 10 vol.% of fibers, i.e., with the lowest percentage of reinforcement, show the fragile behavior of the epoxy matrix, with evidence of river marks and smooth and flat surfaces (Figure 6). Regarding the fibers, transversal (Figure 6a and Figure 7a) and longitudinal (Figure 7a) ruptures, as well as pullout (Figure 7b) were observed.

**Figure 8:**
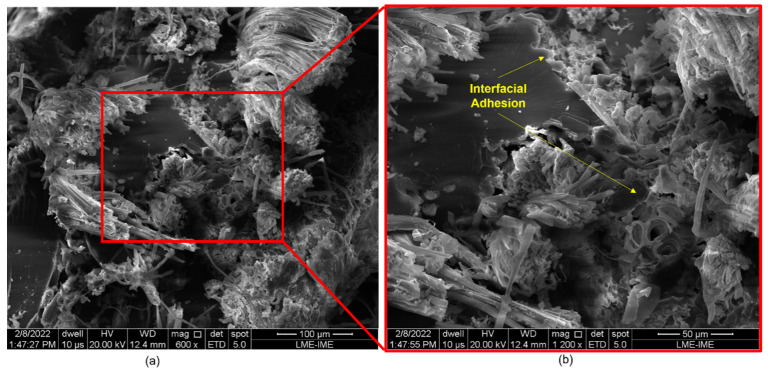
Fracture surface of a composite with 20 vol.% of fibers (PGO/ENT) arranged: unidirectionally (**a**) and bidirectionally (**b**), at 200× magnification.

**Figure 9:**
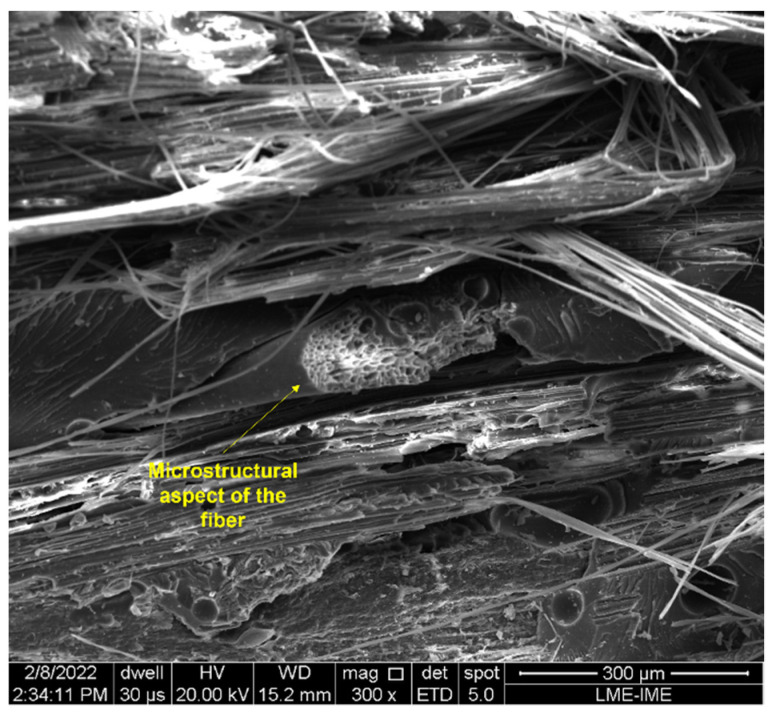
Fracture surface of a bidirectional composite with 30 vol.% fibers (PNT/EGO) at 300× magnification.

**Figure 10:**
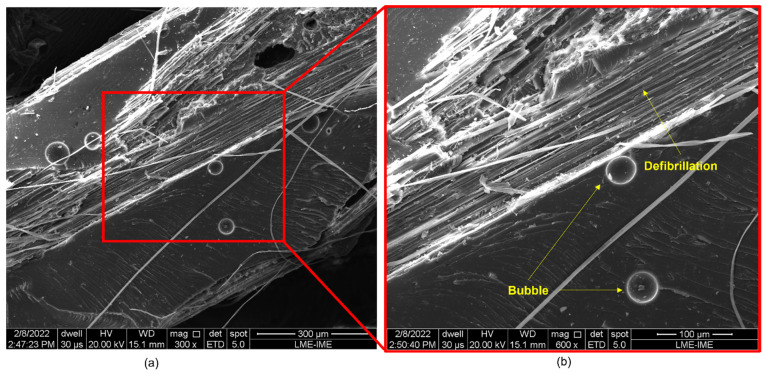
Fracture surface of a composite with 20 vol.% of fibers (PNT/EGO) arranged: unidirectionally (**a**) and bidirectionally (**b**), at 200× magnification.

**Figure 11:**
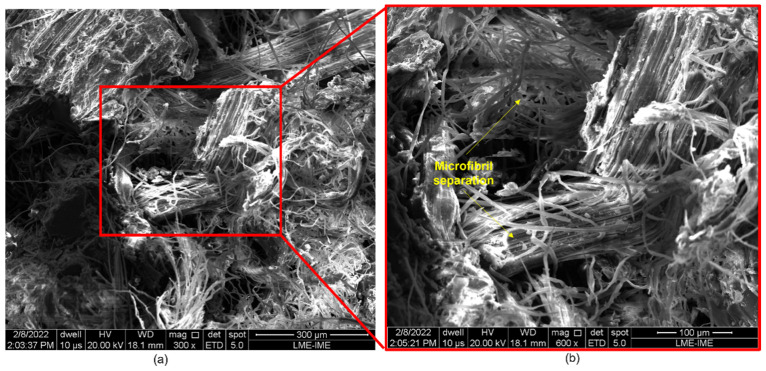
Fracture surface of a bidirectional composite with 30 vol.% fibers (PGO/ENT) at 300× (**a**) and 600× (**b**) magnifications.

**Figure 12:**
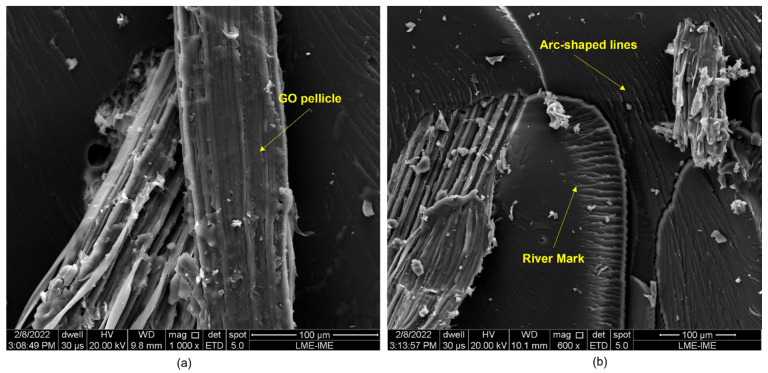
Fracture surface of a bidirectional composite with 30 vol.% fibers (PGO/EGO) at 1000× (**a**) and 600× (**b**) magnifications.

Figure 8, Figure 9, Figure 10, Figure 11 and Figure 12 show the fracture surfaces of GO functionalized composites containing 30 vol.% of PALF, that is, with the highest percentage of reinforcement of the PGO/ENT groups (Figure 8 and Figure 11), PNT/EGO (Figure 9 and Figure 10), and PGO/EGO (Figure 12). As for the failure mechanisms, it is worth noting the fragile fracture of the non-functionalized matrix, good fiber–matrix adhesion, few pullout voids, and ruptured fibers can be seen (Figure 8). Lumens and ruptured fiber cell walls can also be visualized in the cross-section, the surface of fibers not coated with GO (Figure 9). In addition to the failure characteristics of the GO-functionalized epoxy, imperfections (bubbles), and defibrillation (Figure 9 and Figure 10), folded fiber and microfibril separation (Figure 11) are also detected. The general appearance of the surfaces of the samples is also observed in Figure 12a,b, in which the two characteristics of the GO are present, namely the film on the fibers (Figure 12a) and the irregular surface of the matrix (river marks and secondary cracks, arc-shaped lines) (Figure 12b).

Regarding the failure characteristics of the GO-functionalized epoxy, it is worth noting that it does not present an apparently smooth and flat fracture surface as in the non-functionalized epoxy. The epoxy matrix presents a similar appearance to that seen in the micrographs studied by Bortz et al. [70] on fatigue and fracture resistance in GO/Epoxy composites. These authors emphasize that nanometer-sized particles and that fibers cannot explain crack fixation because their relative size is much smaller than the crack tip opening displacement. However, the 2D micrometric dimensions of the GO sheets are thought to be large enough to explain the observation of crack fixation (i.e., surface irregularity).

Arc-shaped lines left on fracture surfaces and secondary cracks associated with river marks are visualized in the micrographs, similar to the previously reported in the literature [46,70]. Bortz et al. [70] pointed out that multiplanar characteristics on the fracture surface of the composite suggest that the GO sheets induce the deflection of the crack front propagation and that this process introduces out-of-plane loading, generating new fracture surfaces, thus increasing the strain energy required for the continuation of fracture.

## 4. Conclusions

Pineapple leaf fibers (PALF)-reinforced epoxy composites tested through the Izod impact test showed a significant increase in absorbed impact energy with the increment of the fiber content, obtaining 287.70 J/m for the untreated composite, containing 30 vol.% of unidirectional PALF. Although this was the highest energy absorbed, it was not considered statistically guaranteed due to the partial rupture of the specimens.

According to the ballistic results, increases in absorbed energy (273.52 ± 67.99 J) and limit velocity (191.95 ± 24.22 m/s) occurred in the composite with 30 vol.% bidirectional untreated PALF and GO-functionalized epoxy matrix (30BD-PNT/EGO). This group presented an increase of 49.17% and 36.19% in absorbed energy, compared to the pure resin and to the untreated group (30BD-PNT/ENT), respectively, which was related to the probable interfacial interactions with the presence of GO and the bidirectional configuration owing to the influence of the reinforcement architecture in more than one direction. The absorbed energy results were verified by ANOVA, confirming a statistically significant difference among the values. Furthermore, an enhancement in the physical integrity of the bidirectional composite plates with the highest volumetric fraction (30BD) was observed. Consequently, the composites studied showed satisfactory performance for ballistic purposes.

The scanning electron microscopy (SEM) observations of the ruptured surfaces after the ballistic test revealed active failure modes for the different groups. These were: river marks, imperfections, pullout, detachment with fiber rupture, rupture of fiber with delamination, microfracture of the matrix, defibrillation, in addition to the fracture characteristics of GO-functionalized composites, such as multiplanar surfaces and secondary cracks associated with river marks in the matrix. The development of such fracture behavior was due to the mechanisms of ballistic energy absorption after the projectile impact.

## Figures and Tables

**Table 1:** Specification of the groups proposed for the ballistic test.

Group	Plate Composition		Fiber Arrangement	GO Functionalization		Label	Number of Plates
	Matrix	Reinforcement		Matrix	Reinforcement		
1	100% Epoxy	0 vol.% PALF	-	No	-	EP-NT	5
2				Yes	-	EP-GO	5
3	90% Epoxy	10 vol.% PALF	Continuous, aligned, and unidirectional	No	No	10UD-PNT/ENT	1
4			Continuous, aligned, and bidirectional	No	No	10BD-PNT/ENT	1
5	80% Epoxy	20 vol.% PALF	Continuous, aligned, and unidirectional	No	No	20UD-PNT/ENT	1
6				Yes	No	20UD-PNT/EGO	1
7				No	Yes	20UD-PGO/ENT	1
8				Yes	Yes	20UD-PGO/EGO	1
9	70% Epoxy	30 vol.% PALF		No	No	30UD-PNT/ENT	1
10				Yes	No	30UD-PNT/EGO	1
11				No	Yes	30UD-PGO/ENT	1
12				Yes	Yes	30UD-PGO/EGO	1
13	80% Epoxy	20 vol.% PALF	Continuous, aligned, and bidirectional	No	No	20BD-PNT/ENT	1
14				Yes	No	20BD-PNT/EGO	1
15				No	Yes	20BD-PGO/ENT	1
16				Yes	Yes	20BD-PGO/EGO	1
17	70% Epoxy	30 vol.% PALF		No	No	30BD-PNT/ENT	1
18				Yes	No	30BD-PNT/EGO	1
19				No	Yes	30BD-PGO/ENT	1
20				Yes	Yes	30BD-PGO/EGO	1

**Table 2:** Tukey test for the Izod impact test absorbed energy values.

Sample	Epoxy	10UD	20UD	10BD	20BD
Epoxy	0	**31.58**	**87.17**	12.35	**71.87**
10UD	**31.58**	0	**55.59**	−19.23	**40.29**
20UD	**87.17**	**55.59**	0	**−74.82**	−15.3
10BD	12.35	−19.23	**−74.82**	0	**59.51**
20BD	**71.87**	**40.29**	−15.3	**59.51**	0

**Table 3:** Izod impact test results from the literature.

Composite	Fiber Configuration and Volumetric Fraction	Absorbed Impact Energy (J/m)	Reference
PALF/Epoxy	Continuous (30%)	503 ± 116.22	[56]
PALF/Epoxy	Continuous (30%)	946.0 ± 140.0	[27]
PALF/LDPE	Random (30%)	≈177	[54]
PALF/Polyester	Continuous (30%)	80.29	[57]
Mallow/Epoxy	Continuous (30%)	498.86	[58]
Tucum/Epoxy	Continuous (40%)	216	[59]
Coconut/Epoxy	Continuous (30%)	111.0 ± 6.8	[27]

**Table 4:** Values acquired from the absorption energy tests.

Samples	$E_{abs}$ (J)	$V_{L}$ (m/s)
EP-NT	183.36 ± 31.39	157.84 ± 14.19
EP-GO	219.20 ± 9.98	172.96 ± 3.92
10UD-PNT/ENT	212.86 ± 34.22	169.91 ± 12.93
10BD-PNT/ENT	203.71 ± 28.44	167.08 ± 11.36
20UD-PNT/ENT	171.20 ± 39.96	152.31 ± 17.61
20BD-PNT/ENT	222.45 ± 17.26	173.83 ± 6.50
30UD-PNT/ENT	179.75 ± 58.57	154.15 ± 27.46
30BD-PNT/ENT	200.83 ± 25.26	165.19 ± 9.90
20UD-PGO/ENT	199.92 ± 15.20	165.03 ± 6.28
20BD-PGO/ENT	218.04 ± 20.92	172.70 ± 8.87
30UD-PGO/ENT	212.66 ± 19.93	170.31 ± 7.75
30BD-PGO/ENT	180.29 ± 57.50	154.73 ± 28.66
20UD-PNT/EGO	201.16 ± 16.57	165.65 ± 6.61
20BD-PNT/EGO	191.48 ± 15.76	161.56 ± 6.62
30UD-PNT/EGO	185.74 ± 31.96	158.69 ± 14.08
30BD-PNT/EGO	273.52 ± 67.99	191.95 ± 24.22
20UD-PGO/EGO	206.15 ± 23.70	167.45 ± 10.08
20BD-PGO/EGO	212.23 ± 17.89	170.16 ± 7.40
30UD-PGO/EGO	194.17 ± 25.54	162.49 ± 10.83
30BD-PGO/EGO	198.63 ± 23.80	164.47 ± 9.46

**Table 5:** Absorbed energy relative to the corresponding projectiles’ masses.

Level (Caliber)	I (0.22)	II (9)	IIIA (44)	IIIA (45)	III (7.62)	
Mass (g)	2.6	8	15.6	≈14.7	9.6	
Samples	*E_abs_* (J)					*E_abs_* (%)
EP-NT	32.59	100.27	195.52	183.36	120.32	44.96
EP-GO	38.9	119.7	233.41	219.2	143.64	54.46
10UD-PNT/ENT	37.8	116.32	226.82	212.86	139.58	51.13
10BD-PNT/ENT	36.45	112.16	218.71	203.71	134.59	47.04
20UD-PNT/ENT	30.49	93.81	182.93	171.2	112.57	41.01
20BD-PNT/ENT	39.32	120.98	235.91	222.45	145.17	42.19
30UD-PNT/ENT	31.68	97.49	190.11	179.75	116.99	35.91
30BD-PNT/ENT	35.59	109.52	213.56	200.83	131.42	43.45
20UD-PGO/ENT	35.43	109.02	212.59	199.92	130.83	48.76
20BD-PGO/ENT	38.83	119.47	232.97	218.04	143.37	53.09
30UD-PGO/ENT	37.77	116.21	226.6	212.66	139.45	51.5
30BD-PGO/ENT	31.98	98.38	191.85	180.29	118.06	44.18
20UD-PNT/EGO	35.73	109.92	214.35	201.16	131.91	49.36
20BD-PNT/EGO	33.98	104.56	203.9	191.48	125.47	46.77
30UD-PNT/EGO	32.94	101.36	197.65	185.74	121.63	45.1
30BD-PNT/EGO	48.48	149.16	290.86	273.52	178.99	66.11
20UD-PGO/EGO	36.54	112.42	219.22	206.15	134.9	49.88
20BD-PGO/EGO	37.69	115.97	226.15	212.23	139.17	51.86
30UD-PGO/EGO	34.44	105.96	206.62	194.17	127.15	47.52
30BD-PGO/EGO	35.28	108.54	211.65	198.63	130.25	48.54

**Table 6:** Absorbed energy values from composites in the literature.

Composite (Matrix—Reinforcement)	Caliber	*E_abs_* (J)	%*E_abs_*	Reference
Epoxy—PALF (30 vol.%)	7.62	169.22 ± 27.50	4.94	[27]
Epoxy—Coconut fiber (30 vol.%)	7.62	190.07 ± 12.08	5.36	[27]
Epoxy—Piassava fiber (20 vol.%)	7.62	272 ± 19	8.07	[5]
Epoxy—Piassava fiber (30 vol.%)	7.62	196 ± 18	5.81	[5]
Epoxy—Piassava fiber (10 vol.%)	7.62	200 ± 15	5.93	[5]
Polyester—Curaua fiber (10 vol.%)	7.62	203 ± 69	6.02	[66]
Polyester—Curaua fiber (20 vol.%)	7.62	163 ± 24	4.84	[66]
Polyester—Curaua fiber (30 vol.%)	7.62	197 ± 25	5.84	[66]
Aramid fabric laminate (16 plies)	7.62	220 ± 17	6.53	[66]
Epoxy—Guaruman fiber (30 vol.%)	0.22	105.5 ± 10.6	78.04	[16]
Epoxy—Sedge fiber (10 vol.%)	0.22	80.5 ± 1.5	57.21	[39]
Epoxy—Sedge fiber (10 vol.%)	0.22	76.3 ± 2.5	54.23	[39]
Epoxy—Sedge fiber (10 vol.%)	0.22	74.0 ± 2.5	52.59	[39]
Epoxy 0,5% GO—Ramie fabric (30 vol.%)	0.22	130.34 ± 9.51	92.63	[44]
(2 mg/mL) GO coated Aramid fabric laminate (5 plies)	9	159.6	29.62	[67]
Aramid fabric laminate (5 plies)	9	106.4	19.75	[67]

**Table 7:** Tukey’s test ADM of the ballistic tests’ mean values.

Sample	20UD-PNT/ENT	30UD-PNT/ENT	30BD-PGO/ENT	EP-NT	30UD-PNT/EGO	20BD-PNT/EGO	30UD-PGO/EGO	30BD-PNT/EGO
20UD-PNT/ENT	0	8.55	9.09	−12.158	14.538	20.276	22.962	**102.31**
30UD-PNT/ENT	8.55	0	0.54	−3.608	5.988	11.726	14.412	**93.76**
30BD-PGO/ENT	9.09	0.54	0	−3.068	5.488	11.186	13.872	**93.22**
EP-NT	−12.158	−3.608	−3.068	0	2.38	8.118	10.804	**90.15**
30UD-PNT/EGO	14.538	5.988	5.488	2.38	0	−5.738	8.424	**87.77**
20BD-PNT/EGO	20.276	11.726	11.186	8.118	−5.738	0	2.686	**82.04**
30UD-PGO/EGO	22.962	14.412	13.872	10.804	8.424	2.686	0	**−79.35**
**30BD-PNT/EGO**	**102.31**	**93.76**	**93.22**	**90.15**	**87.77**	**82.04**	**−79.35**	0

## Data Availability

Not applicable.
